# p52 expression enhances lung cancer progression

**DOI:** 10.1038/s41598-018-24488-8

**Published:** 2018-04-17

**Authors:** Jamie A. Saxon, Hui Yu, Vasiliy V. Polosukhin, Georgios T. Stathopoulos, Linda A. Gleaves, Allyson G. McLoed, Pierre P. Massion, Fiona E. Yull, Zhongming Zhao, Timothy S. Blackwell

**Affiliations:** 1 Department of Cancer Biology, Vanderbilt University, Nashville, TN 37232 USA; 2 Department of Biomedical Informatics, Vanderbilt University School of Medicine, Nashville, TN 37203 USA; 3 Center for Quantitative Sciences, Vanderbilt University Medical Center, Nashville, TN 37232 USA; 4 Department of Medicine, Division of Allergy, Pulmonary and Critical Care Medicine, Vanderbilt University, Nashville, TN 37232 USA; 5 Comprehensive Pneumology Center (CPC) and Institute for Lung Biology and Disease (iLBD), University Hospital, Ludwig-Maximilian University (LMU) and Helmholtz Center Munich, Member of the German Center for Lung Research (DZL), Max-Lebsche-Platz 31, 81377 Munich, Bavaria Germany; 6 Laboratory for Molecular Respiratory Carcinogenesis, Department of Physiology, Faculty of Medicine, University of Patras, 1 Asklepiou Str., 26504 Rio, Achaia Greece; 7 Department of Veterans Affairs Medical Center, Nashville, TN 37232 USA; 8 Department of Cell and Developmental Biology, Vanderbilt University, Nashville, TN 37232 USA

## Abstract

While many studies have demonstrated that canonical NF-κB signaling is a central pathway in lung tumorigenesis, the role of non-canonical NF-κB signaling in lung cancer remains undefined. We observed frequent nuclear accumulation of the non-canonical NF-κB component p100/p52 in human lung adenocarcinoma. To investigate the impact of non-canonical NF-κB signaling on lung carcinogenesis, we employed transgenic mice with doxycycline-inducible expression of p52 in airway epithelial cells. p52 over-expression led to increased tumor number and progression after injection of the carcinogen urethane. Gene expression analysis of lungs from transgenic mice combined with *in vitro* studies suggested that p52 promotes proliferation of lung epithelial cells through regulation of cell cycle-associated genes. Using gene expression and patient information from The Cancer Genome Atlas (TCGA) database, we found that expression of p52-associated genes was increased in lung adenocarcinomas and correlated with reduced survival, even in early stage disease. Analysis of p52-associated gene expression in additional human lung adenocarcinoma datasets corroborated these findings. Together, these studies implicate the non-canonical NF-κB component p52 in lung carcinogenesis and suggest modulation of p52 activity and/or downstream mediators as new therapeutic targets.

## Introduction

Lung cancer is the leading cause of cancer-related death in the U.S, with an estimated 224,390 new lung cancer diagnoses in 2016 and a 5-year survival rate of less than 20 percent^1^. In addition to emphasizing the need for better understanding of tumor biology and development of new therapeutic approaches, available epidemiological data suggest that identification of biomarkers for improved outcome prediction beyond the current staging system would be valuable for guiding appropriate use of available treatment strategies.

Numerous studies have demonstrated the critical role of epithelial NF-κB signaling in lung cancer^2–4^. The NF-κB transcription factor family contains 5 members (p65/RelA, p52, p50, RelB, and c-Rel) and can be activated through either canonical or non-canonical signaling pathways. Canonical pathway activation occurs when inhibitory IκBs are phosphorylated, releasing p65/p50 heterodimers to translocate into the nucleus. Non-canonical NF-κB signaling is defined by nuclear accumulation of p52, which hinges on proteolytic processing of p100 removing an inhibitory C-terminal domain and resulting in p52 activation. p52 is then able to enter the nucleus, typically as a heterodimer bound to RelB. Although components of both the canonical and non-canonical NF-κB signaling pathways are activated in a number of malignancies, including lung cancer^5–7^, studies of NF-κB in cancer have mainly focused on canonical pathway signaling, leaving the non-canonical pathway largely uninvestigated.

The majority of knowledge regarding *in vivo* functions of p100/p52 relate to its role in lymphoid development and hematopoietic disease. Mice with global knockout of the *Nfkb2* gene, which encodes p100, demonstrate defective dendritic cell function, B-cell maturation, T-cell responses, and secondary lymphoid organ development^8,9^, presumably due to the role of p52 in regulating expression of chemokine genes important for normal lymphoid organogenesis^10^. In several hematopoietic malignancies, including multiple myeloma, chronic lymphocytic leukemia, and B- and T-cell cutaneous lymphomas, chromosomal translocations in the *Nfkb2* gene have been identified that truncate the C-terminus, thus removing the inhibitory domain^11–13^. These truncated proteins localize in the nucleus, leading to constitutively active p52^11^. Knock-in mice that express truncated *Nfkb2* develop enlarged lymph nodes and hyperactive T cell responses, providing some insight into the function of activated p52 in hematopoietic diseases^14^. However, these mice die prematurely due to significant gastric hyperplasia, hinting that dysregulated p52 activation could also play a role in epithelial-based disorders.

Increased activation of p52 has been observed in lung^6^, breast^15^, prostate^16,17^, and pancreatic cancers^18^; however, studies on the effects of p52 activation in cancers of epithelial origin have been limited due to lack of appropriate *in vivo* models. Here, we use a transgenic mouse model with inducible expression of p52 in the airway epithelium (designated CCSP-p52) to investigate a potential role for p52 in lung tumorigenesis. Together, these studies define a role for p52 in lung tumorigenesis and identify a p52-associated gene network that is highly correlated with patient prognosis.

## Results

### p100/p52 expression is common in human lung adenocarcinoma

**Figure 1:**
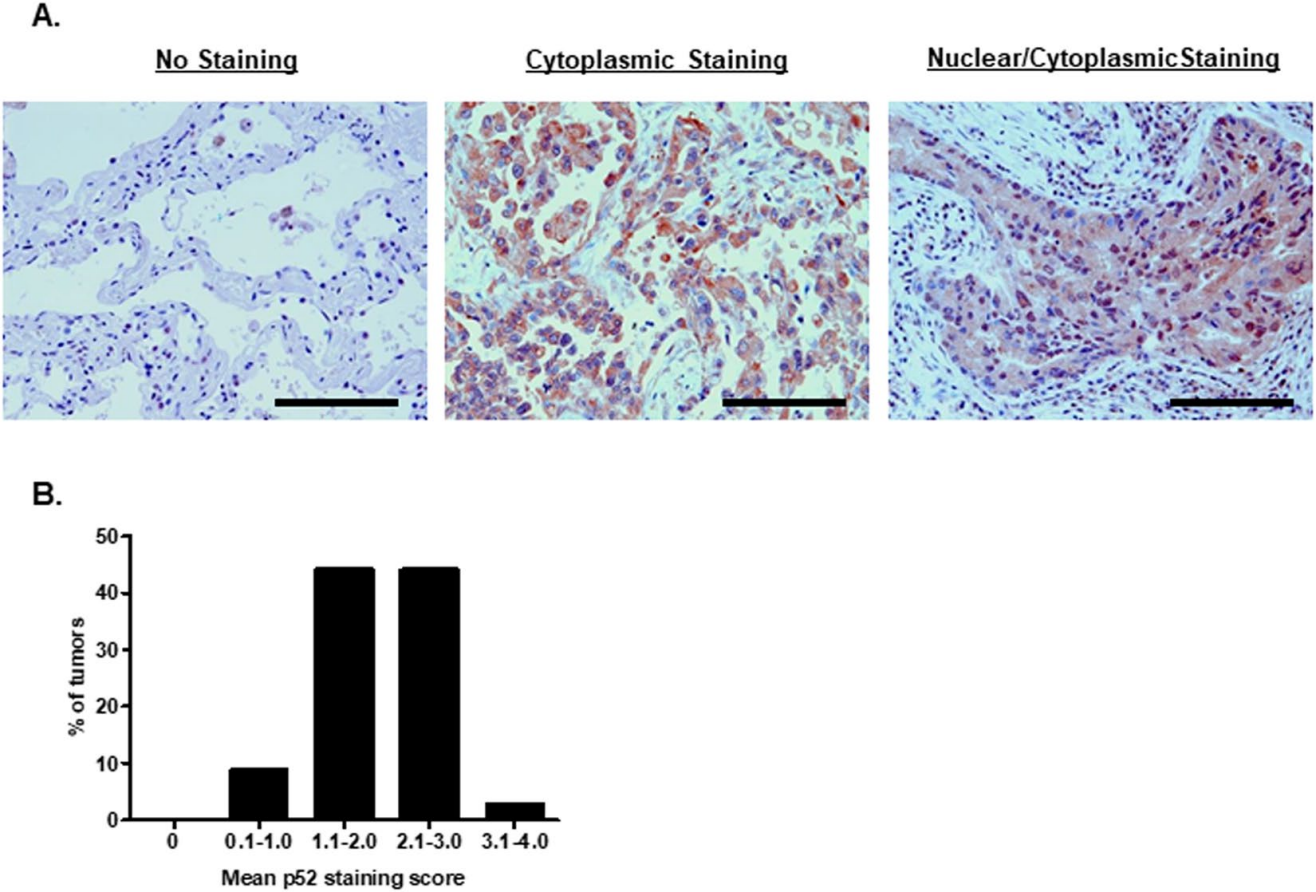
p100/p52 expression is present in human lung adenocarcinomas. (**A**) Representative images of p100/p52 immunostaining of lung adenocarcinoma and normal lung tissue (40x magnification; scale bar = 100 µm). (**B**) Distribution of p100/p52 immunostaining on a tumor tissue microarray (TMA) from 106 human lung adenocarcinoma samples obtained from 34 patients. Tumor spots were scored as follows: 0 = no staining; 1 = diffuse cytoplasmic staining, no nuclear staining; 2 = dark granular cytoplasmic staining, no nuclear; 3 = nuclear staining in <25% of tumor cells; 4 = nuclear staining in >25% of tumor cells. Each tumor had at least two spots on the TMA, and the mean score was calculated for each tumor based on the individual score for each spot.

To examine the distribution of p100/p52 expression in human lung adenocarcinoma, we performed p100/p52 immunostaining on a lung adenocarcinoma tissue microarray (TMA) (Fig. 1A, Table 1). We observed p100/p52 expression in all lung adenocarcinomas tested (34/34) (Fig. 1B). Multiple samples from each tumor were present on the array, and each sample was scored on a 0 to 4 point scale based on the intensity and the localization of p100/p52 staining. We calculated an average p100/p52 expression score for each tumor and found that 47% (16/34) of tumors had mean scores > 2, indicating substantial nuclear staining for p52. In contrast, minimal p100/p52 expression was observed in normal lung parenchyma (Fig. 1A). Together, these observations suggest that p100/p52 expression and p52 activation are common in lung adenocarcinomas.

**Table 1:** Clinicopathological characteristics of patient samples.

Factor	n	Mean ± SE
Age (years)		
≤60	12	54.41 ± 1.31
>60	21	67.95 ± 0.99
Unknown	1	
Ever Smoker		
Yes	32	
No	1	
Unknown	1	
Pack Years		
≤50	16	34.99 ± 2.33
>50	15	109.95 ± 11.90
Unknown	1	
Stage at Diagnosis		
Stage I (A and B)	22	
Stage II (A and B)	5	
Stage III (A and B)	5	
Unknown	2	

### CCSP-p52 mice develop an increased tumor burden and more advanced lesions

In order to investigate the effect of p52 expression during lung tumorigenesis, we employed CCSP-p52 mice, which inducibly express FLAG-tagged murine p52 in Clara cell secretory protein (CCSP)-positive epithelial cells^19^. These mice exhibit normal lung histology after p52 over-expression^19^. To induce tumors in CCSP-p52 mice, we used the lung carcinogen urethane (ethyl carbamate), which causes tumors primarily through *Kras* mutations^20^ and, in FVB background mice, induces a mild inflammatory response^2^. We first examined the effects of p52 over-expression on urethane-induced inflammation and atypical adenomatous hyperplasia (AAH) formation. For these experiments, CCSP-p52 and wild-type (WT) mice were treated with dox for one week followed by a single intraperitoneal (IP) injection of urethane (1 g/kg) and remained on dox until euthanized. Total inflammatory cells in bronchoalveolar lavage (BAL) were counted at 10 days, 21 days (3 weeks), and 42 days (6 weeks) after urethane injection. No differences were observed between CCSP-p52 and WT mice at any time point (Supplementary Fig. S1A). In addition, no differences in AAH lesion numbers were detected at 6 weeks post-urethane (Supplementary Fig. S1B).

**Figure 2:**
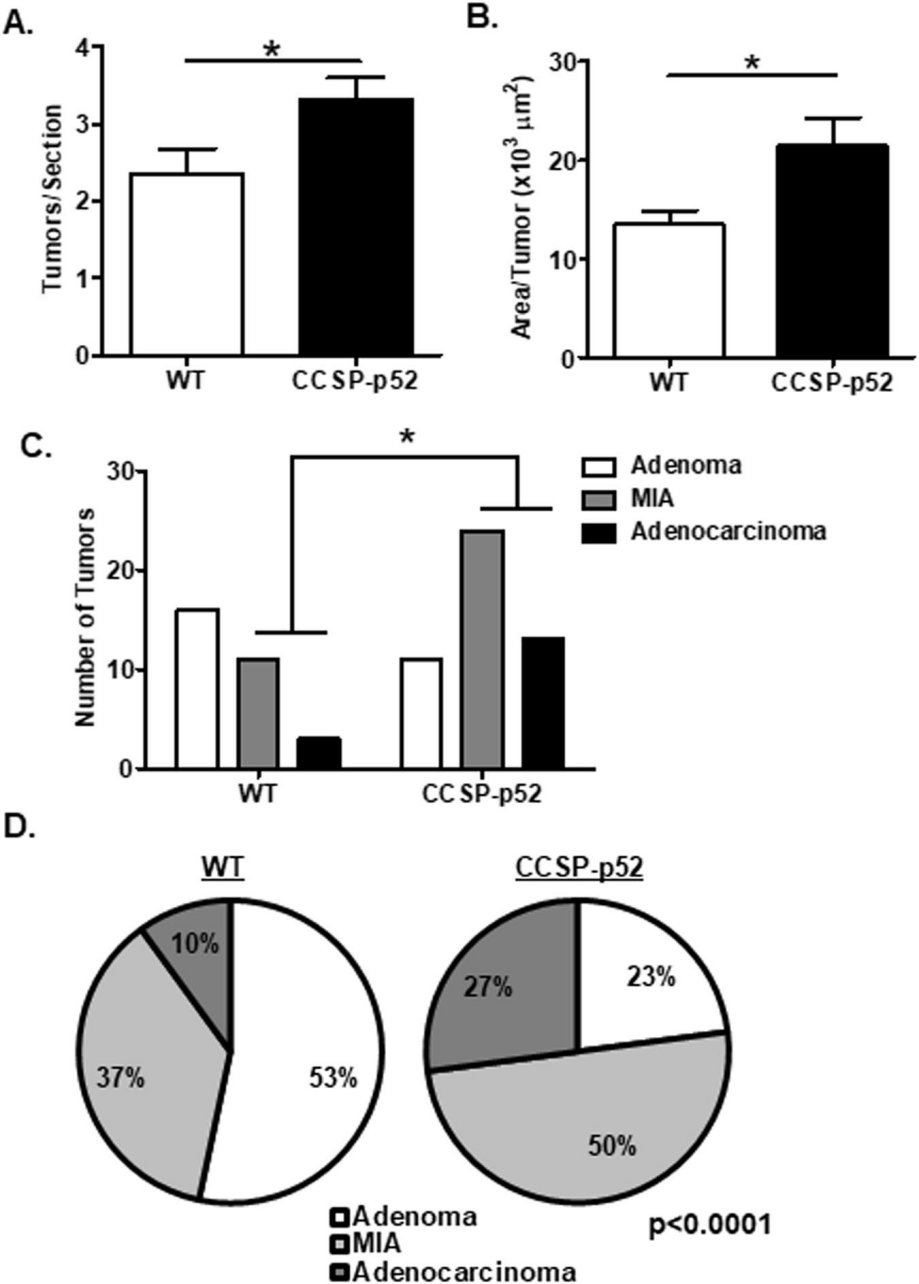
p52 over-expression results in increased tumor number, size, and progression. (**A**) Average tumor number per lung section and (**B**) average tumor area from WT and CCSP-p52 mice 6 months after urethane injection (3 sections/mouse; n = 13–14 mice/group; *p < 0.05 compared to WT mice.). (**C**) Number and (**D**) proportion of lung tumors classified as adenomas, minimally invasive adenocarcinomas (MIA), or adenocarcinomas (n = 13–14 mice/group; *Χ^2^ p < 0.0001 for comparing total number of advanced lesions [MIAs and adenocarcinomas] between CCSP-p52 and WT mice).

To investigate later stages of tumor formation, CCSP-p52 and WT mice were treated with dox for one week followed by 4 weekly IP urethane injections. Mice remained on dox treatment until sacrifice 6 months after the first urethane injection. Lung tumor dimensions were measured, and lung tumors enumerated on lung sections, revealing a significant increase in tumor number and tumor size in CCSP-p52 lungs compared to WT (Fig. 2A and B). We further analyzed these tumors by histology, classifying tumors as adenomas, minimally invasive adenocarcinomas (MIAs), or adenocarcinomas. Compared to WT mice, CCSP-p52 mice had an increased proportion of advanced lesions (MIAs and adenocarcinomas, Χ^2^ p < 0.0001) (Fig. 2C and D). In comparison, long-term p52 expression alone resulted in sustained p52 expression but no evidence of inflammation or tumor formation (Supplementary Fig. S2). Taken together, these studies demonstrate that p52 expression augments lung tumor formation and contributes to enhanced tumor progression.

### p52 expression enhances proliferation through regulation of cell cycle genes

**Figure 3:**
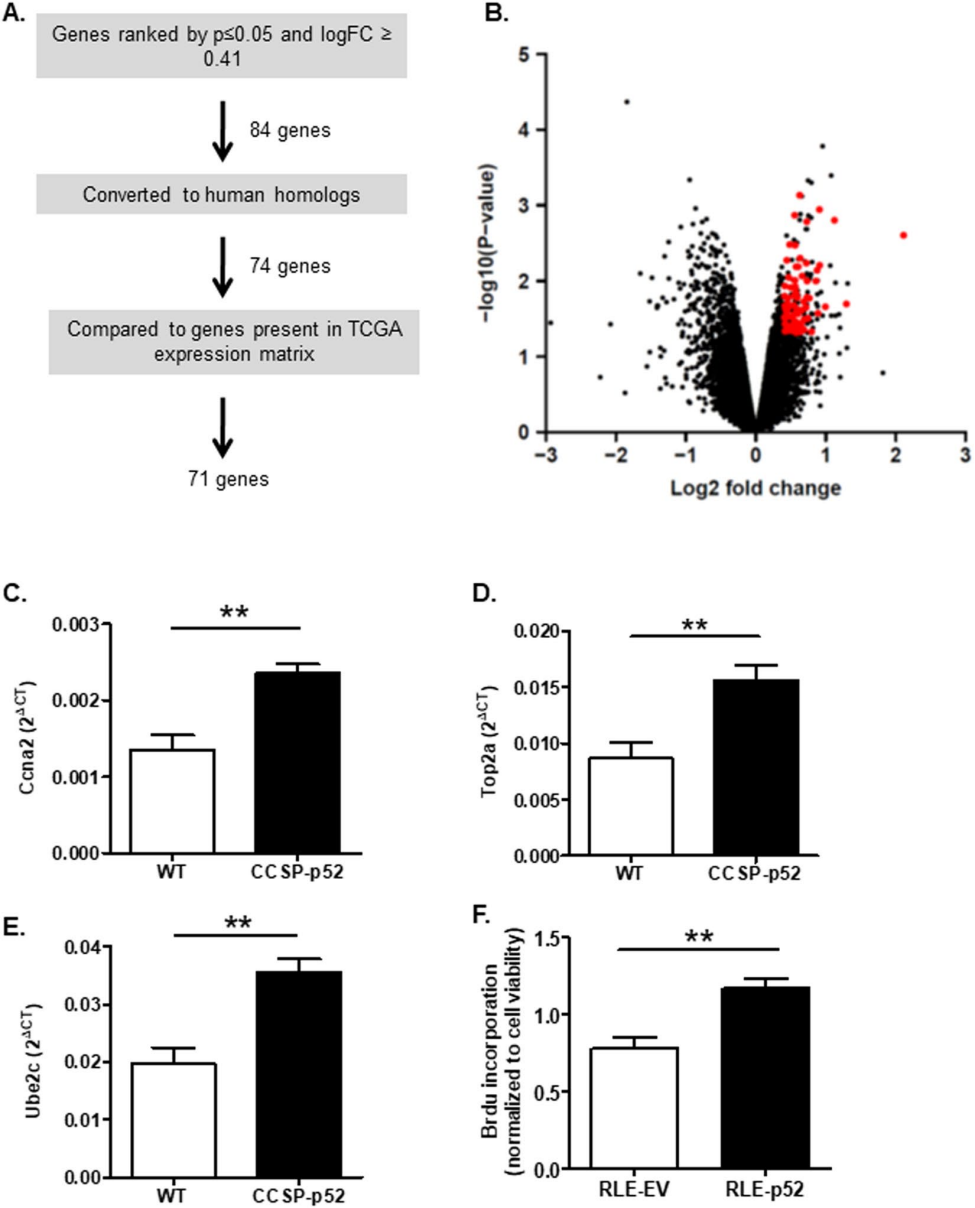
Identification of p52-regulated genes by microarray analysis. (**A**) Schematic of process used to identify p52-regulated genes from microarray data generated from pooled mRNA samples from lungs of WT and CCSP-p52 mice on dox for 1 week (n = 4/group). (**B**) Volcano plot of log fold changes and differential expression p-values comparing CCSP-p52 mice to WT. Black dots represent all genes in the expression data matrix. Red dots represent 71 genes identified for further analysis. (**C**–**E**) Expression microarray data validation by quantitative PCR of identified p52-regulated genes *Ccna2* (**C**), *Top2a* (**D**), and *Ube2c* (**E**) measured in whole lung RNA from WT and CCSP-p52 mice on dox for 1 week (n = 6 mice/group; *p < 0.05 compared to WT). (**F**) Quantification of BrdU incorporation of RLE-p52 and RLE-WT cells, normalized to total viable cells (**p < 0.01 compared to RLE-EV).

To understand the mechanism by which p52 expression augments tumorigenesis, we performed microarray gene expression analysis using mRNA isolated from lungs of CCSP-p52 and WT mice on dox for 1 week. Genes were ranked based on the log fold change (logFC) of expression between WT and CCSP-p52 mice using a cut-off of 0.41 (approximately 30% increase in gene expression in CCSP-p52 mouse lungs) (Fig. 3A). To evaluate the potential relevance of p52-regulated genes in human lung tumors, we focused only on genes that had known human homologs and were present in The Cancer Genome Atlas (TCGA) expression data from lung adenocarcinoma patients. Using these criteria, a total of 71 genes associated with increased p52 expression were identified (Fig. 3A and B; Supplementary Table S1). Quantitative PCR was performed to validate increased expression of selected genes from the 71 gene list in CCSP-p52 lungs (Fig. 3C–E). To elucidate gene functions, we identified biological processes that were enriched in the p52-associated gene set using a Gene Ontology (GO) analysis. Applying a false discovery rate (FDR) of 0.001, we found that p52-associated genes were over-represented in processes related to cell cycle progression (Table 2), suggesting that p52 regulates expression of genes involved in proliferation. To validate this finding, we used a stable p52-expressing rat lung epithelial cell line (RLE-p52) generated previously^19^. We found that BrdU incorporation was enhanced in RLE-p52 cells compared to empty vector (RLE-EV) (Fig. 3F), indicating that p52 stimulates proliferation of lung epithelial cells.

**Table 2:** Gene ontology (GO) analysis of p52 regulated genes (FDR = 0.001).

Gene Ontology term (Biological Process)	Reference set size	Expected Count	Observed count	Adjusted p-value^1^
Cell cycle checkpoint	230	0.98	9	2.59E-05
Mitotic cell cycle phase transition	443	1.88	11	8.83E-05
DNA unwinding involved in DNA replication	8	0.03	3	1.39E-04
Regulation of cell division	221	0.94	8	1.39E-04
Regulation of attachment of spindle microtubules to kinetochore	9	0.04	3	1.81E-04
DNA replication initiation	29	0.12	4	1.88E-04
Regulation of cell cycle process	435	1.85	10	3.68E-04
Chromosome organization	765	3.25	13	4.04E-04
Spindle assembly involved in mitosis	15	0.06	3	7.44E-04

^1^p-values have been adjusted using the Benjamini-Hochberg method.

### Expression of p52-associated genes predicts prognosis of lung cancer patients

**Figure 4:**
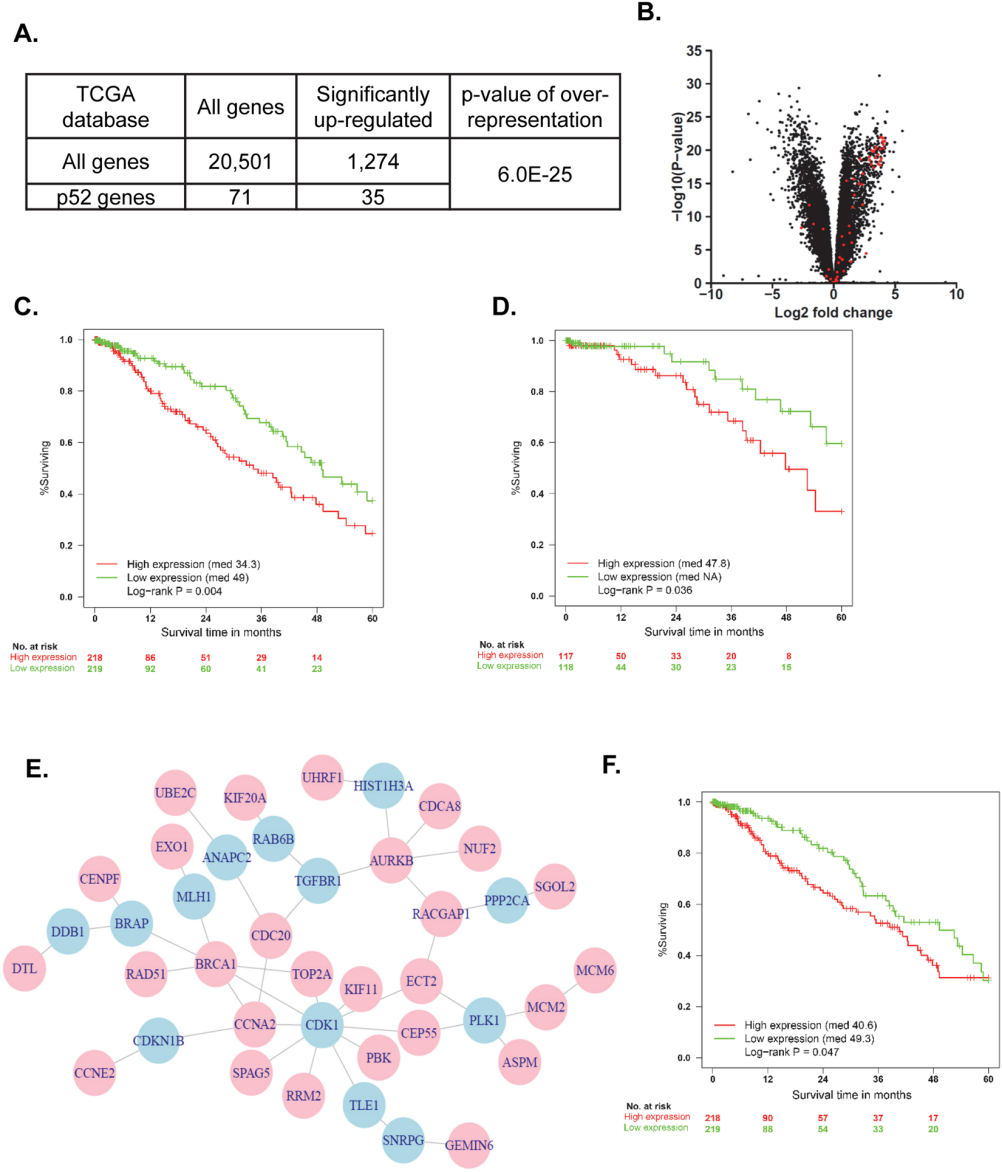
Expression of p52-associated genes correlates with prognosis of lung cancer patients. (**A**) Global and p52-associated genes upregulated in matched tumor and normal samples (n = 54) from TCGA dataset. Up-regulated genes were defined as logFC > 1 and FDR < 0.01 (hypergeometric test p = 6.0 × 10^−25^). (**B**) Volcano plot of log fold changes and differential expression p-values of tumor versus normal samples. Black dots represent all genes in the expression data matrix. Red dots represent p52-associated genes. (**C**,**D**) Kaplan-Meier survival curves of overall (**C**) and Stage I (**D**) patient survival data based on summed expression of p52-associated genes in tumors (log-rank test, p = 0.004 for overall; p = 0.036 for Stage I). (**E**) Protein-protein interaction network generated using 35 p52-associated genes associated with tumors (see Fig. 4A and Supplementary Table S1). Pink circles are genes identified as p52-regulated genes through microarray analysis, and blue circles are imputed genes. (**F**) Kaplan-Meier survival curve of overall patient survival based on summed expression of imputed mediator genes (log-rank test, p = 0.047).

To investigate the relevance of p52 expression in human lung cancer, we examined expression of the 71 identified p52-associated genes in matched lung cancer and normal samples. A differential expression test using the paired t-test was performed for each gene in paired tumor and normal samples from 54 lung adenocarcinoma patients available in the TCGA dataset. Up-regulated genes were defined as having both a logFC > 1 and false discovery rate (FDR) < 0.01. From the set of 20,501 genes, 1,274 up-regulated genes (6.2%) were identified in tumors compared to matched normal samples (Fig. 4A). Of the 71 p52-associated genes, 35 were up-regulated in tumors (49.3%), indicating a significant over-representation of p52-associated genes in tumor samples compared to normal (hypergeometric test p = 6.0 × 10^−25^) (Fig. 4A,B and Supplementary Table S1) and suggesting that p52 could be important in human lung tumors.

Since we observed more advanced tumors in urethane-treated CCSP-p52 mice, we wondered whether increased p52 expression would correlate with a worse prognosis in lung cancer patients. Therefore, we investigated the relationship between increased expression of p52-associated genes and patient outcomes using tumor expression data and clinical information from the TCGA dataset. 437 primary lung adenocarcinoma samples were divided into two equal groups based on the collective expression level of the 71 p52-associated genes by summing expression from all 71 genes (Supplementary Table S2). Kaplan-Meier survival analysis revealed that patients with high expression of p52-associated genes had a significantly worse prognosis (log-rank test p = 0.004) (Fig. 4C). To validate this analysis, we divided patients by a second method into two groups based on summed “votes” from the 71 genes. In this analysis, one gene counted as a vote for one sample if its expression in the sample was equal to or higher than the median expression level for this gene across samples, thus preventing a tumor sample with only a few highly-expressed genes from being placed in the “high expression group”. Using this method, Kaplan-Meier survival analysis again revealed that patients with high expression of p52-associated genes had significantly worse outcomes (log-rank test p = 0.021) (Supplementary Fig. S3A). Furthermore, survival analysis of only Stage I patients revealed that patients with high expression of p52-associated genes still had a significantly worse prognosis (log-rank test p = 0.036) (Fig. 4D). Together, these data suggest that expression of p52-associated genes in tumors of lung cancer patients leads to shorter survival and can be used to predict patient outcome in early stage lung cancer patients.

Since the GO analysis suggested that p52 regulates a number of genes that have highly related functions, we extracted a protein subnetwork connecting the p52-associated genes from a human protein-protein interaction network. The 35 p52-associated genes identified in tumor versus normal samples were used to generate a network consisting of genes from the p52-associated gene list as well as 13 imputed mediator genes (Fig. 4E). To test the strength of this protein interaction network, a survival analysis was performed based only on expression of the 13 imputed mediator genes. High expression of the imputed genes also correlated with shorter survival (log-rank test p = 0.047 by expression summation, p = 0.097 by votes) (Fig. 4F and Supplementary Fig. S3B), supporting the strength of this p52-derived interaction network in predicting patient prognosis.

**Figure 5:**
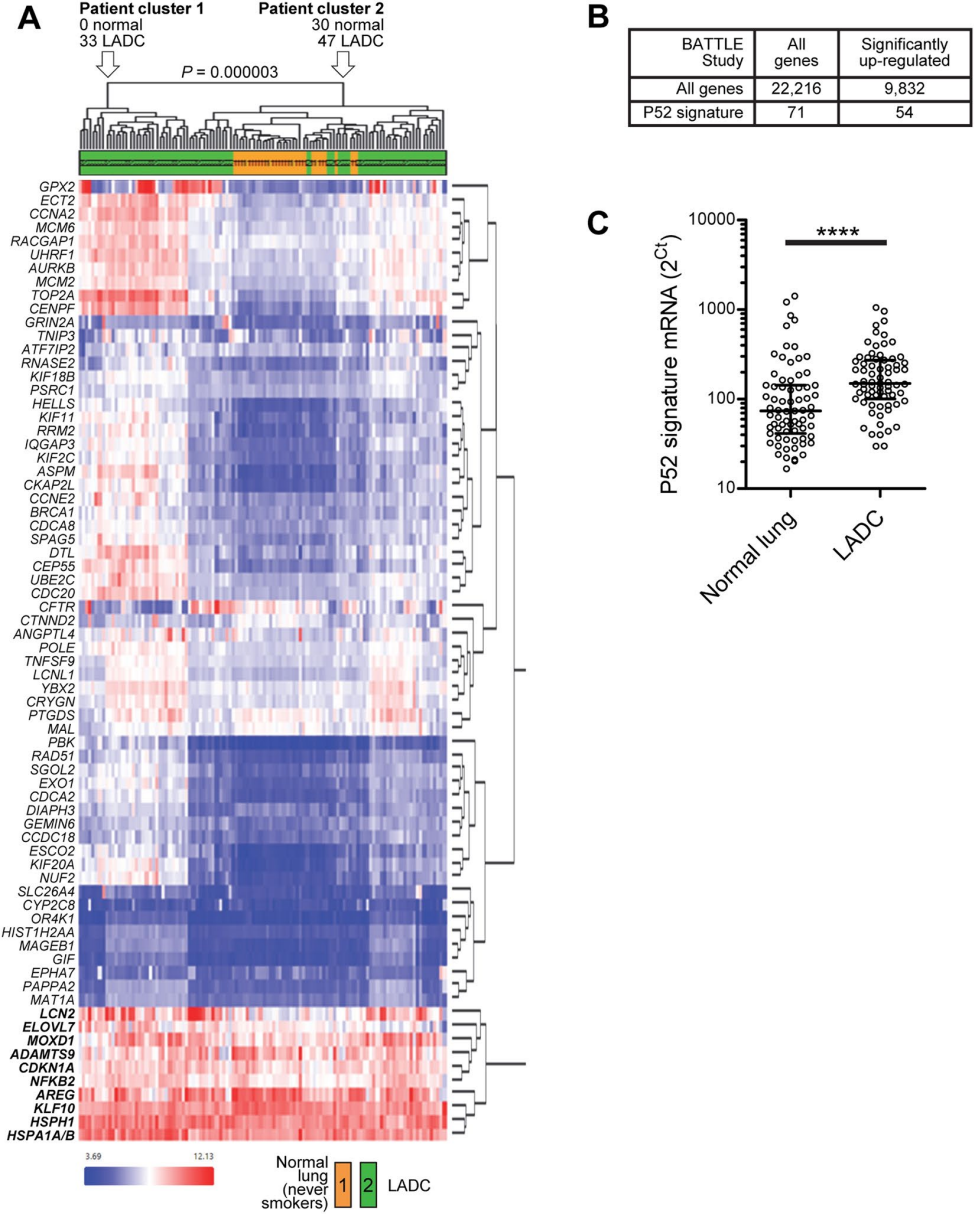
Increased expression of p52-associated genes in lung adenocarcinoma samples from the BATTLE study. (**A**) Hierarchical clustering based on expression of 71 p52-associated genes in normal lung (n = 30) and lung adenocarcinoma (LADC) (n = 80) tissues. (Fisher’s exact test p = 0.000003). (**B**) p52 signature and global genes over-represented in lung adenocarcinoma samples compared with normal lung. Up-regulated genes were defined as logFC > 1.3 and FDR < 0.01 (Fischer’s exact test P = 0.0034). (**C**) mRNA transcript expression levels from 71 p52-associated genes in normal lung tissue and lung adenocarcinoma samples. Raw data points and median with error bars that represent interquartile range are shown. Transcripts were not normally distributed by the Kolmogorov-Smirnov normality test and were hence analyzed by the Wilcoxon matched-pairs signed rank test (****p < 0.0001).

We further validated our original 71 p52-associated gene signature in additional human lung adenocarcinoma datasets. Using normal and lung adenocarcinoma expression data from the BATTLE study^21,22^, we performed unsupervised hierarchical clustering and found that this p52-associated gene signature segregated normal and lung adenocarcinoma samples (Fisher’s exact test p = 0.000003) (Fig. 5A). We then performed a differential expression test for each gene in normal and lung adenocarcinoma samples, defining up-regulated genes as having both a logFC > 1.3 and FDR < 0.01. From the entire set of 22,216 genes, 9,832 (44.3%) were significantly upregulated in tumors compared to matched normal samples whereas 54 (76.1%) of the 71 p52-associated genes were up-regulated in tumors, demonstrating a significant over-representation of p52-associated genes in tumor samples compared to normal in a second independent dataset (Fisher’s exact test p = 0.0034) (Fig. 5B). When evaluated as a group, mRNA levels of the 71 p52-associated genes were significantly increased in lung adenocarcinoma samples compared to normal lung tissue (p < 0.0001) (Fig. 5C). In addition, we assessed the relationship between p52 expression and survival of lung adenocarcinoma patients in a composite dataset that includes expression data from several lung cancer cohorts^23^. Higher p100/p52 (*Nfkb2*) expression in lung adenocarcinomas correlated with reduced patient survival (p = 3 × 10^−7^) (Supplementary Fig. S4B). In contrast, higher expression of *Nfkb1*, which encodes for the NF-κB family member p50, correlated with improved survival (p = 0.017) (Supplementary Fig. S4A).

## Discussion

These studies provide evidence of the functional involvement of non-canonical NF-κB signaling in lung carcinogenesis, identifying p52 as a novel mediator of lung cancer progression. In human lung adenocarcinoma tumors, we observed frequent nuclear accumulation of p52, suggesting that p52 activation is common in lung carcinogenesis. Using transgenic mice with inducible expression of p52 in the airway epithelium, we found that p52 over-expression led to more tumors, larger tumors, and more advanced tumors after urethane injection. Our *in vitro* studies and gene expression microarray analysis suggest that p52 enhances tumor burden through the regulation of cell cycle genes that promote proliferation. Expression of p52-associated genes was significantly associated with lung tumors and increased expression of genes from a p52-derived protein-protein interaction network correlated with significantly reduced survival, even for Stage I patients. These findings have important implications for investigations into modulation of p52 or its downstream targets as a novel therapeutic approach in lung cancer.

Even though nuclear localization of p52 has been observed in a number of different tumor types, our understanding of the function of p52 activation *in vivo* during carcinogenesis has remained limited due to the constraints of available models. In other studies using a mouse model with global truncation of the *Nfkb2* gene deleting the C-terminal inhibitory domain of p100, mice die from gastric hyperplasia^14^. However, unlike our studies, the individual contribution of inflammatory cells and surrounding stroma with dysregulated p52 activation was not separated from the effects of p52 activation in the epithelial cells of the stomach. Using a transgenic model with expression of the full *Nfkb2* gene under the control of the β-lactoglobulin milk protein promoter, Connelly *et al*. demonstrated ductal thickening and hyperplasia in the mammary gland after repeated pregnancies^24^, but in this model, expression was limited to the periods of milk production during the late stages of pregnancy and lactation. Our studies improved upon these models by using an inducible p52-expressing mouse. The advantages of a mouse model with inducible p52 expression are that p52 can be expressed with any cell type-specific promoter coupled to a reverse tetracycline transactivator, the duration of p52 expression can be manipulated, and p52 expression can be combined with other inducible mouse models, including oncogenic tumor models. Future studies using this p52-inducible model to modulate the duration of expression in the urethane model and to express p52 in other cell types and in combination with other inducible models will be valuable for developing a broader understanding of p52 activation in lung cancer and other contexts.

Although p100/p52 expression appears to be common in lung adenocarcinomas, how this pathway becomes activated remains unclear. Proteolytic processing of p100 to p52 can be activated by a number of stimuli, including lymphotoxin β^10^, CD40 ligand^25^, reactive oxygen species^26^, and STAT3 signaling^27^, suggesting that p52 activation can occur in a tumor as a result of oncogenic signaling or microenvironmental stimuli. Additional evidence indicates that crosstalk occurs between the canonical and non-canonical NF-κB pathways, suggesting that factors leading to canonical pathway activation could also activate p52^28^.

Like CCSP-p52 mice, activation of canonical NF-κB signaling in transgenic mice expressing constitutively active IKKβ in airway epithelium increases tumor burden following urethane treatment^29^. In this model, canonical NF-κB activation causes a robust immune/inflammatory response, which promotes lung carcinogenesis through paracrine signaling^29,30^. In contrast, our studies indicate that non-canonical NF-κB signaling likely regulates genes that function in a cell autonomous manner to promote carcinogenesis.

Our finding that p52 can promote proliferation of airway epithelial cells during lung carcinogenesis is somewhat unexpected, as several previous studies investigating p52 expression in airway epithelial cells have shown that p52 can promote apoptosis of Club cells *in vivo*^19^ and contribute to regulation of inflammatory cytokines *in vitro*^31^. In other studies using *in vitro* and xenograft models, however, p52 activation has been shown to promote cell proliferation. Nadiminty *et al*. observed that p52 over-expression enhanced proliferation of prostate cancer cells in androgen-deprived conditions by stimulating Cyclin D1 expression^32^. Similarly, increased p100 expression in mammary glands led to hyperplasia and increased Cyclin D1^24^. Although we did not identify Cyclin D1 as an upregulated gene in our microarray analysis, both Cyclin E2 and Cyclin A2 were increased in CCSP-p52 lungs (Supplementary Table S1).

Among NF-κB family members, p52 is structurally most similar to p50. Kravtsova-Ivantsiv and colleagues found that p50 over-expression inhibits tumor growth in xenograft models^33^, and our survival analysis based on p50 expression in lung adenocarcinomas supports their observations. Despite the structural similarity between p50 and p52, we found that p52 over-expression enhances tumorigenesis and leads to reduced survival. These studies suggest that p52 and p50 function differently during tumorigenesis, which may be due to the differential affinities of each for specific promoter sequences or their propensity to interact with other NF-κB family members and transcriptional binding partners^34,35^. While nuclear p52 accumulation is considered indicative of non-canonical NF-κB pathway activation, a study by Zhao, *et al*. suggests that p52 is capable of forming heterodimers with all of the other NF-κB family members^34^. In addition, p52 has been shown to interact with Bcl-3^36–39^, p53^40^, and multiple epigenetic modifiers, including CBP, p300, and HDAC proteins^38,40–43^, and these interactions can be activating or inhibitory depending on the context. Future studies are necessary to understand p52 interactions in the nucleus and the mechanisms by which p52 influences tumor formation and progression.

In these studies, we took a gene list derived from p52 over-expression in a mouse model and demonstrated that these genes predict survival of early stage lung cancer patients. In addition, p52 expression in lung adenocarcinomas was inversely correlated with survival, similar to previous reports with the NF-κB family members p65 and RelB^44,45^. Although differences in gene expression between CCSP-p52 and WT mouse lungs were relatively modest, likely due to the small subset of transgene-expressing lung epithelial cells, we were able to identify genes with significant expression changes as a result of p52 over-expression, and these genes were highly related in function, indicating that p52 regulates expression of a network of related genes. Using gene expression profiling of *Nfkb2* knockdown fibroblasts, Ianetti *et al*. described corresponding changes in several of the same genes identified in our study, supporting our identification of a discrete set of p52 target genes^26^. However, future studies comparing p52-induced gene expression changes during different stages of carcinogenesis and in the context of different oncogenic stimuli could be helpful for revealing additional p52 target genes.

Since our data suggest that p52 regulates a network of known cell cycle regulators that have well-established roles in carcinogenesis, p52 modulation may be an attractive therapeutic target for drug development. Although there are no available methods to directly target p52 at present, inhibitors for several of the nodes in our p52-derived protein interaction network have been developed and are being tested in clinical trials, including aurora kinase B, cyclin dependent kinase 1, and polo-like kinase 1, suggesting that treatment with these drugs alone or in combination may be beneficial in patients with p52 activation in their tumors.

## Materials and Methods

### Animal model

CCSP-tTS/(tet-O)_7_-FLAG-p52 mice on an FVB background were bred to CCSP-rtTA homozygous mice (gift from Dr. J.A. Whitsett, University of Cincinnati, Cincinnati, OH) to generate CCSP-p52 mice, which inducibly express FLAG-tagged p52 in the airway epithelium as previously described^19^. CCSP-p52 mice generated from two separate CCSP-tTS/(tet-O)_7_-FLAG-p52 founder lines were used for these studies. Age- and sex-matched transgenic mice along with genotype-negative littermate controls (called WT in our studies) were used in experiments. Transgene expression was activated by administering doxycycline (dox, Sigma) at a concentration of 2 g/L along with 2% sucrose in drinking water. For urethane experiments, mice were placed on dox for 1 week prior to IP injection of urethane (1 g/kg body weight; Sigma-Aldrich) and remained on dox until euthanized. For 6 month tumorigenesis experiments, mice received weekly IP injections of urethane for 4 weeks. At sacrifice, lungs were lavaged, and total and differential BAL cell counts were determined as previously described^2,29^. The left lung was tied off and frozen, and the right lung was perfused and fixed by inflating with 10% neutral-buffered formalin. All animal studies were approved and conducted according to the guidelines of the Vanderbilt University Medical Center Institutional Animal Care and Use Committee.

### Histology and immunostaining

After fixation, lungs were embedded in paraffin, sectioned (5 µm), and hematoxylin and eosin (H&E)-stained for histological analysis and quantification of tumor burden. Three sections were analyzed per mouse, and each section was separated by 50 µm. Tumor area was measured using Image-Pro Plus software (Media Cybernetics), and tumor number was enumerated on each section. AAH lesions and tumor histology were assessed by a pathologist blinded to the experimental groups. Tumors were classified as adenomas (clear border), minimally invasive adenocarcinomas (MIAs; border mostly defined with one invasive edge), and adenocarcinomas (locally invasive with no defined border). To assess p100/p52 expression in human lung adenocarcinomas, immunostaining using a p100/p52 antibody (C-5, Santa Cruz) was performed on a lung tumor tissue microarray (TMA) with 106 adenocarcinoma samples (spots) from 34 patients. Collection of tumor samples for generating the TMA was carried out in accordance with relevant guidelines and regulations, and experimental protocols were approved by the Vanderbilt University Medical Center Institutional Review Board (IRB 000616). Informed consent was obtained from all subjects. p100/p52 staining of tumor spots was scored by two independent readers. Scoring was performed as follows: 0 = no staining; 1 = diffuse cytoplasmic staining, no nuclear staining; 2 = dark granular cytoplasmic staining, no nuclear; 3 = nuclear staining in <25% of tumor cells; 4 = nuclear staining in >25% of tumor cells. Each tumor had at least two spots on the TMA, and the mean score was calculated for each tumor based on the individual score for each spot.

### Western blot analysis

Nuclear protein was prepared from lung tissue using the NE-PER Nuclear and Cytoplasmic Extraction Reagents (Thermo Scientific), separated by SDS-PAGE gel, transferred to nitrocellulose membranes, and probed using p100/p52 (4882, Cell Signaling) and nuclear loading control TATA-binding protein (TBP) (N-12, Santa Cruz). Immunodetection was performed using the corresponding AlexaFluor-conjugated antibodies and the Odyssey Infrared Imaging System (LI-COR Biosciences). All images were converted to grayscale.

### *In vitro* proliferation measurements

Rat type II alveolar epithelial cell line RLE-6TN was obtained from ATCC and maintained at 37 °C 5% CO_2_ in DMEM (Invitrogen) with 4.5 g/l glucose and 2 mM L-glutamine, supplemented with 10% fetal bovine serum, 100 units/ml penicillin, and 100 µg/ml streptomycin. Cells were tested periodically for mycoplasma contamination using the Universal Mycoplasma Detection Kit (ATCC). Generation and validation of stable p52 over-expressing (RLE-p52) and control empty vector (RLE-EV) cells are described elsewhere^19^. RLE-EV and RLE-p52 cells were plated in 96 well plates for viability and BrdU incorporation measurements. 48 hours after plating, cells were incubated with BrdU for 4 hours, and BrdU incorporation was measured using the chemiluminescent BrdU Cell Proliferation ELISA (Roche). To ensure plating of equal numbers of cells, viability of RLE-EV and RLE-p52 cells was measured on the same day as a surrogate for cell number using the CellTiter-Glo Luminescent Cell Viability assay (Promega) according to the manufacturer’s protocol.

### Microarray gene expression analysis

The RNeasy Mini kit (Qiagen) was used to isolate whole lung RNA from the lungs of CCSP-p52 and WT mice treated with dox for 1 week and with or without intratracheal LPS administration, which was performed as part of a separate study [described in ref.^19^]. To minimize the effects of mouse-to-mouse-variability, RNA was pooled from 3 mice for each sample, resulting in a total of 8 samples (2 WT dox only, 2 WT with LPS, 2 CCSP-p52 dox only, 2 CCSP-p52 with LPS). RNA quality control, hybridization to the Affymetrix Mouse Gene 1.0 ST array, and array scanning were performed by the Vanderbilt Technologies for Advanced Genomics (VANTAGE) Core. Expression data have been deposited in Gene Expression Omnibus (Accession # GSE71648).

The Robust Multichip Average method^46^ implemented in R package “oligo” (v1.28.3)^47^ was employed to normalize imported raw data. Probe sets interrogating control, unmapped, or intron sequences were ignored, leaving 79% of probe sets for a differential expression analysis. Linear models and empirical Bayes methods implemented in R package “limma” (v3.20.8)^48^ were applied to estimate log fold changes and p-values for the p52-vs-WT effect. Since samples with and without LPS stimulation were processed and microarray profiled at the same time, we assumed the random data variations were similar across all samples and built a linear model across the eight samples accounting for both p52 and LPS experimental factors^49^. The coefficient (estimated log fold change) and p-value associated with the p52 factor were retrieved for the selection of differentially expressed genes. This method enabled us to isolate the specific effects of p52 and LPS, allowing us to focus only on genes changed by p52 over-expression and effectively giving us 4 samples per group (12 mice per group) for our gene expression comparisons. When selecting top-ranking p52-induced genes, only probe sets mapped to known genes with positive expression changes were considered.

### Quantitative PCR

Quantitative real-time PCR was performed using Sybr Green PCR Master Mix (Applied Biosystems) and the following primer sets: Ccna2 F: AAGAGAATGTCAACCCCGAAAAA; R: ACCCGTCGAGTCTTGAGCTT (PrimerBank ID: 161353443c1), Top2a F: CAACTGGAACATATACTGCTCCG; R: GGGTCCCTTTGTTTGTTATCAGC (PrimerBank ID: 6755849a1), Ube2c F: CTCCGCCTTCCCTGAGTCA; R: GGTGCGTTGTAAGGGTAGCC (PrimerBank ID 21312888a1) and Gapdh (F: TGAGGACCAGGTTGTCTCCT R: CCCTGTTGCTGTAGCCGTAT)^50–52^. Expression values were normalized to Gapdh using the ΔCT method.

### Statistics

Unpaired student t-tests were performed for comparisons between two groups. Values are presented as the mean ± SEM, and p ≤ 0.05 was considered statistically significant. Data were analyzed using GraphPad Prism 5.0 software (GraphPad Software, Inc.). Analysis of transcriptomic data is presented below.

### Human lung cancer expression data analysis

Raw expression data for 513 lung adenocarcinoma patient samples, generated by the TCGA project, were accessed from the ICGC data portal (https://dcc.icgc.org/; release 17). The raw RSEM values^53^ were multiplied by 10^6^ to normalized values of Transcripts Per Million (TPM). Of these 513 samples, matched tumor samples and paracancerous normal samples were available for 54 patients. A paired t-test was performed for each gene, and the resultant p-values were adjusted to false discovery rates using the Benjamini-Hochberg method^54^.

Of the 513 lung adenocarcinoma samples, 437 primary lung adenocarcinoma samples had accompanying survival outcome data. In each survival analysis, samples were divided into two groups of equal sizes (termed high-expression and low-expression groups) by considering the collective expression of p52-associated genes. R package “survival” (v2.37-7)^55^ was employed to conduct survival analyses. In survival analyses, survival time values were truncated to 5 years (1825 days) to reflect 5-year survival rates.

For the BATTLE study analysis, global gene expression profiles of normal lung and lung adenocarcinoma, as assessed by Human Gene 1.0 ST microarrays (Affymetrix, Sta. Clara, CA), were retrieved from Gene Expression Omnibus series GSE43458^21,22^. Raw data were analyzed using Affymetrix Gene Expression and Transcriptome Analysis Consoles.

### Gene functional analysis

Functions enriched within p52-regulated genes were identified through a hypergeometric test with respect to GO terms^56^, using R package GOstats (v1.7.4)^57^. For each GO term, genes from the reference set of whole human genome protein-coding genes and the concerned set of p52-regulated genes were respectively identified, and a p-value indicative of enrichment significance was calculated and further adjusted to the FDR using the Benjamini-Hochberg method^54^. Resultant GO terms were pruned to lowest common ancestors according to the Biological Process semantic topology.

From the Human Protein Reference Database (HPRD, v9)^58^, a connected protein interaction network was derived that comprised 9,218 nodes and 36,728 edges. p52-regulated genes were mapped to this HPRD network, and then the Steiner minimal tree algorithm^59^ was implemented to retrieve the most parsimonious subnetwork that joined together the terminal genes.

## Electronic supplementary material

Supplementary Figures
