# Supplementary material for: p52 expression enhances lung cancer progression
Source: Sci Rep. 2018 Apr 17;8:6078. doi: 10.1038/s41598-018-24488-8 (PMC5904214; doi:10.1038/s41598-018-24488-8)
Supplement: Supplementary file 1 — Supplementary Figures [file 41598_2018_24488_MOESM1_ESM.doc]

**p52 expression enhances lung cancer progression**

Jamie A. Saxon, Hui Yu, Vasiliy V. Polosukhin, Georgios T. Stathopoulos, Linda A. Gleaves, Allyson G. McLoed, Pierre P. Massion, Fiona E. Yull, Zhongming Zhao, and Timothy S. Blackwell

**Supplementary Figure S1:**

**a.**

**b.**

**Supplementary Figure S1:** p52 expression does not affect urethane-induced inflammatory cell recruitment or formation of atypical adenomatous hyperplasia (AAH). A) Total inflammatory cells in BALs from WT and CCSP-p52 mice 10 days, 21 days (3 weeks), and 42 days (6 weeks) after urethane injection (10 and 21 day n=7-8 mice/group; 42 day n=22-24 mice/group). B) Average number of AAH lesions counted on lung sections from WT and CCSP-p52 mice 6 weeks after urethane (n=13-16 mice/group).


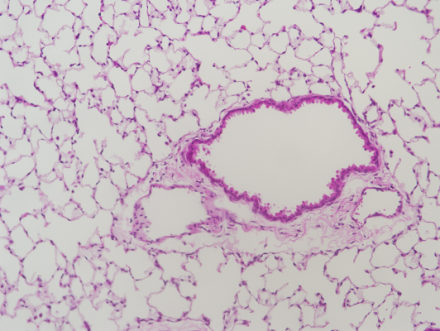

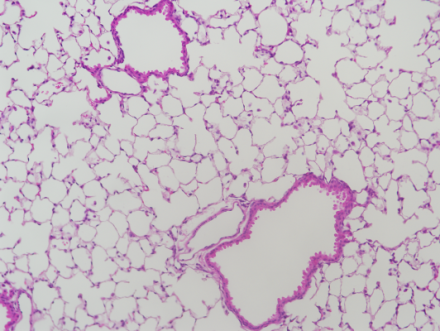


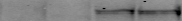

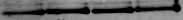


**p52**

**TBP**

**CCSP-p52**

**WT**

**Supplementary Figure S2:**

**a.**

**b.**

**c.**

**WT**

**CCSP-p52**

**Supplementary Figure S2:** Long-term p52 expression does not affect inflammatory cell recruitment or lung histology. A) Western blot demonstrating p52 transgene expression in lung nuclear protein from WT and CCSP-p52 mice on dox for 6 months. TBP was probed as a nuclear loading control. B) Total BAL inflammatory cell numbers and C) representative lung photomicrographs (20x magnification) from WT and CCSP-p52 mice (n=8 mice/group).


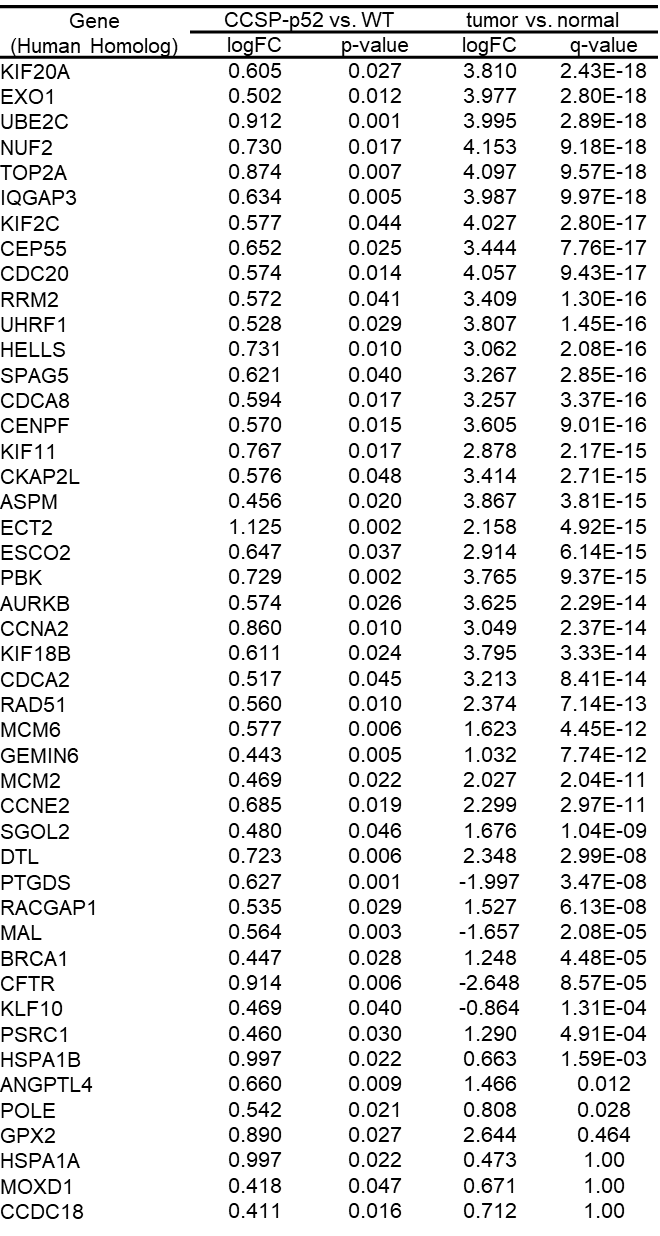


**Supplementary Table S1:**

| CRYGN | 0.421 | 0.037 | 282.976 | 1.00 |
| --- | --- | --- | --- | --- |
| MAGEB1 | 0.425 | 0.023 | 193.612 | 1.00 |
| LCN2 | 0.585 | 0.036 | 1.387 | 1.00 |
| HSPH1 | 0.689 | 0.033 | 0.379 | 1.00 |
| PAPPA2 | 0.608 | 0.007 | -176.187 | 1.00 |
| CTNND2 | 0.547 | 0.035 | -126.328 | 1.00 |
| CDKN1A | 0.432 | 0.025 | -0.415 | 1.00 |
| CYP2C8 | 0.417 | 0.022 | -106.253 | 1.00 |
| MAT1A | 0.409 | 0.031 | 0.815 | 1.00 |
| NFKB2 | 0.720 | 0.022 | 0.279 | 1.00 |
| GRIN2A | 0.416 | 0.012 | -70.563 | 1.00 |
| HIST1H2AA | 0.545 | 0.016 | 52.794 | 1.00 |
| TNIP3 | 2.112 | 0.003 | -0.598 | 1.00 |
| AREG | 0.565 | 0.012 | -0.599 | 1.00 |
| DIAPH3 | 0.441 | 0.025 | 0.303 | 1.00 |
| ADAMTS9 | 0.564 | 0.040 | -0.261 | 1.00 |
| YBX2 | 0.556 | 0.001 | 91.762 | 1.00 |
| ATF7IP2 | 0.443 | 0.025 | 0.167 | 1.00 |
| SLC26A4 | 1.295 | 0.020 | 0.262 | 1.00 |
| GIF | 0.485 | 0.003 | 35.537 | 1.00 |
| EPHA7 | 0.573 | 0.035 | -17.780 | 1.00 |
| TNFSF9 | 0.796 | 0.047 | 0.093 | 1.00 |
| ELOVL7 | 0.455 | 0.017 | -0.075 | 1.00 |
| RNASE2 | 0.742 | 0.031 | 1.143 | 1.00 |
| OR4K1 | 0.471 | 0.009 | 0.070 | 1.00 |


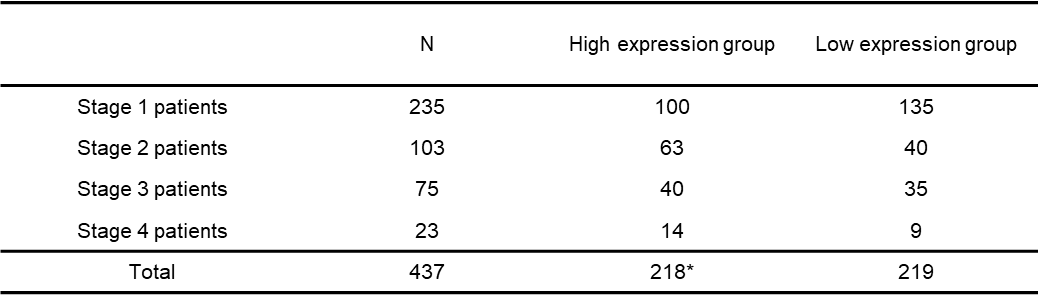


**Supplementary Table S2:**

*One sample in the high expression group had data for survival and expression but no stage information. This sample was included in overall analysis but not in stage-specific analysis.


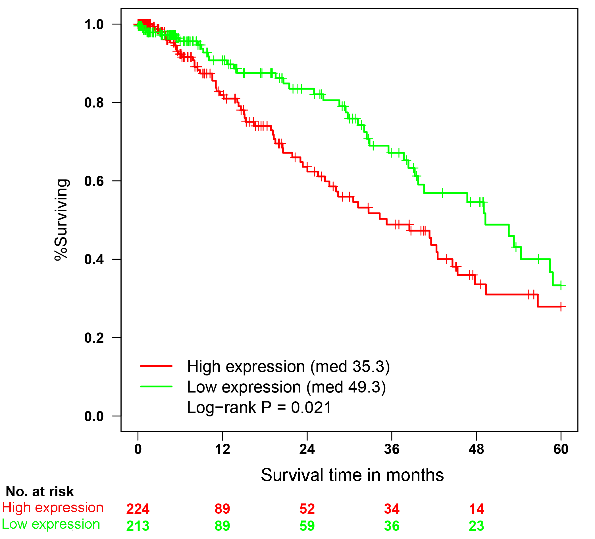

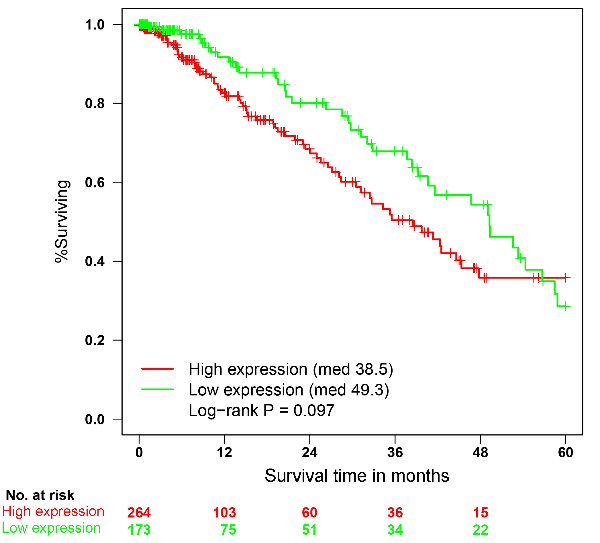


**Supplementary Figure S3:**

**B.**

**A.**

**Supplementary Figure S3:** Expression of p52-associated genes based on “voting” method correlates with poor patient outcomes. A) Kaplan-Meier survival curve of overall patient survival data divided based on expression “votes” of p52-associated genes (log-rank test, p=0.021). B) Kaplan-Meier survival curve of overall patient survival based on expression “votes” of imputed mediator genes (log-rank test, p=0.097).


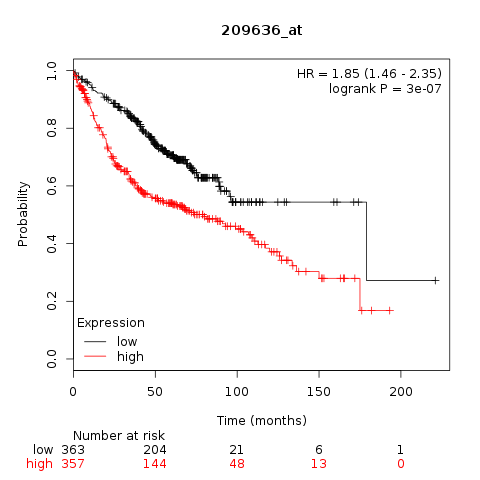

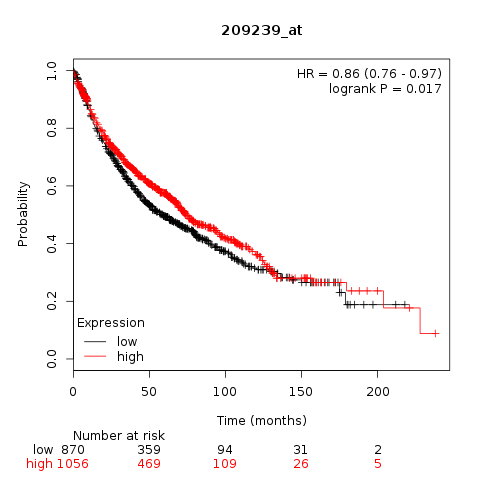


**Supplementary Figure S4:** p52 expression negatively impacts survival in human lung adenocarcinoma. Kaplan-Meier survival analysis of patients with lung adenocarcinoma (*n* = 720) stratified by (a) *Nfkb1* (encoding p50; optimal cut-off = 852 relative mRNA abundance units) and (b) *Nfkb2* (encoding p52; optimal cut-off = 65 relative mRNA abundance units) expression levels. Data were obtained from http://kmplot.com/ (Gyorffy B, Surowiak P, Budczies J, Lanczky A. Online survival analysis software to assess the prognostic value of biomarkers using transcriptomic data in non-small-cell lung cancer, PLoS One, 2013 Dec 18;8(12):e82241. doi: 10.1371/journal.pone.0082241).

**A**

**B**

**Supplementary Figure S4:**

v

v
